# Explainable Artificial Intelligence in Dentistry: A Systematic Review of Its Trust and Translation

**DOI:** 10.1016/j.identj.2026.109626

**Published:** 2026-05-25

**Authors:** Sermporn Thaweesapphithak, Vivat Thongchotchat, Hamid Alinejad-Rokny, Lakshman Samaranayake, Thanaphum Osathanon, Thantrira Porntaveetus

**Affiliations:** a Center of Excellence in Precision Medicine and Digital Health, Department of Physiology, Faculty of Dentistry, Chulalongkorn University, Bangkok, Thailand; b Department of Oral Biomedical Sciences, International College of Dentistry, Walailak University, Bangkok, Thailand; c UNSW BioMedical Machine Learning Lab (BML), School of Biomedical Engineering, UNSW Sydney, Sydney, NSW 2052, Australia; d Visiting Scholar (Collaborative Projects), Center of Excellence in Precision Medicine and Digital Health, Chulalongkorn University, Bangkok, Thailand; e Faculty of Dentistry, University of Hong Kong, Hong Kong; f Global Research Cell, Dr. D. Y. Patil Dental College and Hospital, Dr. D. Y. Patil Vidyapeeth, Pimpri, Pune, India; g Center of Excellence for Dental Stem Cell Biology, Center of Artificial Intelligence and Innovation, Department of Anatomy, Faculty of Dentistry, Chulalongkorn University, Bangkok, Thailand; h Chulalongkorn University Implant and Esthetic Center, Faculty of Dentistry, Chulalongkorn University, Bangkok, Thailand; i Clinic of General-, Special Care and Geriatric Dentistry, Center for Dental Medicine, University of Zurich, Zurich, Switzerland

**Keywords:** Explainable AI, Interpretable machine learning, Dentistry, Systematic review, Trust, Clinical adoption, QUADAS-2, PROBAST

## Abstract

**Introduction and aims:**

Explainable artificial intelligence (XAI) is a set of methods and processes that make the decisions of artificial intelligence (AI) models understandable to those who are not conversant with the technology. This “black box” nature of complex AI models appears to be a primary barrier to their clinical adoption in health sciences, including dentistry. XAI is being touted as a solution to build clinician trust. This review critically assesses whether current dental XAI research is methodologically rigorous enough to substantiate claims of enhanced trustworthiness.

**Methods:**

This systematic review followed Preferred Reporting Items for Systematic Reviews and Meta-Analyses 2020 guidelines, searching PubMed, IEEE Xplore, medRxiv, and Ovid for dental XAI studies (2015-2025). We assessed the risk of bias and applicability using QUADAS-2 and PROBAST.

**Results:**

Nineteen of the 100 identified studies met the inclusion criteria. Although these studies used diverse XAI techniques, including image-based saliency methods (eg, Grad-CAM), feature attribution approaches (eg, SHAP), and local approximation methods (eg, LIME) across various dental specialties, quality assessment exposed significant limitations. Most (14 of 19) exhibited a high risk of bias, driven by small retrospective datasets, lack of external validation, and weak reference standards. Interestingly, only 1 study has evaluated the impact of XAI on human understanding.

**Conclusion:**

Current dental XAI research remains in a proof-of-concept phase, characterised by technical demonstrations based on low-quality evidence. The field has not yet substantiated claims that XAI enhances clinical trust or decision-making. To bridge this gap, future work must prioritise methodological rigour, external validation, and, most importantly, human-centred evaluations with dental professionals to measure the true impact of explainability on clinical workflows and patient care.

**Clinical Relevance:**

Current dental XAI lacks the evidence quality required for clinical reliance. Practitioners should exercise caution, as these tools have not been proven to actually improve diagnostic accuracy or trust in daily practice. Until validated in real-world settings, XAI remains experimental technology rather than a standard of care.

## Introduction

Artificial intelligence (AI) is poised to revolutionise dentistry, offering advancements in diagnostic imaging, treatment planning, and outcome prediction.^1^, ^2^, ^3^ However, the clinical integration of these technologies, particularly complex deep learning models, is hampered by their so-called black box nature. When an AI system provides a diagnosis without a transparent rationale, it undermines clinician trust, complicates regulatory approval, and poses ethical challenges for patient care.^1^^,^^4^, ^5^, ^6^

Explainable artificial intelligence (XAI) has therefore emerged as a critical field aimed at making AI decisions interpretable to humans.^7^^,^^8^ A suite of techniques, from saliency maps like Grad-CAM (a gradient-based heatmap method for imaging) for imaging to model-agnostic methods like SHAP (feature attribution based on game theory) and LIME (local approximation of model behaviour) for tabular data, promises to open these “black boxes”.^9^^,^^10^ In a clinical context, the purported value of XAI is not merely technical transparency but the fostering of trust, allowing a clinician to verify, understand, and confidently act upon an AI’s recommendation.^11^^,^^12^

The expanding application of XAI to dental problems is clear, yet the mere provision of an explanation is an insufficient foundation for clinical trust. An explanation that rationalises a flawed or biased prediction is not just unhelpful; it is potentially dangerous. Consequently, the pivotal issue is not the presence of XAI but the quality of its implementation within methodologically robust research. From a clinical perspective, this translates to a single, pragmatic concern: “Was the AI system developed and validated with the rigour necessary to make its explanations both reliable and actionable for dental professionals?”

This systematic review moves beyond cataloguing applications to critically appraise the evidence base for XAI in dentistry. We synthesise the current landscape but focus our analysis on a central thesis: the path to trustworthy clinical AI in dentistry requires more than just technical explanations; it demands that those explanations be built upon methodologically rigorous and clinically evaluated models.

To operationalise this objective, established methodological frameworks were applied with distinct and complementary roles. Preferred Reporting Items for Systematic Reviews and Meta-Analyses (PRISMA) guidelines were used to ensure transparent, reproducible, and unbiased reporting of the review process itself.^11^ QUADAS-2 was applied to diagnostic accuracy studies to evaluate whether XAI explanations were grounded in diagnostically valid models with appropriate patient selection, reference standards, and evaluation pathways.^13^ PROBAST was used to assess prediction model studies, allowing examination of whether XAI-supported predictions were built upon models with acceptable risk of bias, robustness, and clinical applicability.^14^^,^^15^ Together, these frameworks were employed not as procedural requirements but as analytical tools to identify the gap between technically explainable AI outputs and the level of evidence required for meaningful clinical trust and adoption.

## Methods

This review was conducted in accordance with the PRISMA 2020 statement.^16^ A completed PRISMA 2020 checklist is provided in Supplementary File 1. The protocol was designed to answer the following question: “What is the state of evidence for Explainable AI in dental diagnostics, and to what extent do current studies demonstrate the methodological rigour necessary for fostering clinical trust?” The protocol was submitted to PROSPERO (CRD420251182324) (https://www.crd.york.ac.uk/PROSPERO/view/CRD420251182324).

### Eligibility criteria

We included original studies that:

1. Applied a XAI technique (eg, saliency maps, Grad-CAM, SHAP, LIME, attention mechanisms, rule-based systems) or provided proxy explainability in the form of AI-generated, clinician-interpretable visual or textual cues directly linked to individual predictions
2. Focused on a dental or oral health diagnostic task
3. Were published in English between January 1, 2015, and August 30, 2025

We excluded reviews, editorials, conference abstracts without full text, and studies where the AI was not directly applied to a dental context or where explainability was not a central component.

### Definition and taxonomy of explainability in dental AI

To ensure a consistent and transparent classification of explainability across the included studies, a priori taxonomy was established and applied during study selection and data extraction. This framework draws from established conceptual models in the XAI literature,^7^^,^^17^ categorising systems into 2 primary groups: XAI and non-XAI.

XAI (recognised and proxy): this category includes AI systems that provide supplementary information to support clinician understanding of model outputs. It encompasses recognised approaches that offer direct insight into model decision-making, such as saliency-based visualisations, feature attribution methods, and inherently interpretable models,^7^^,^^18^ as well as systems that provide clinician-interpretable cues at the user-interface level, including visual highlights, bounding boxes, or textual markers linked to individual predictions. While interface-level cues may not be derived from explicit model attribution mechanisms, they were classified as proxy explainability because they contextualise model outputs and facilitate clinical interpretation without revealing the underlying reasoning of the model.^9^^,^^19^

AI systems that provide only prediction outputs, such as binary classifications or probability scores, without accompanying explanatory cues, visualisations, or clinician-facing justifications, are classified as nonexplainable AI systems.^7^ These models do not provide insight into the factors underlying their predictions and were therefore considered opaque from an explainability perspective.

### Information sources and search strategy

A systematic search was performed in PubMed, IEEE Xplore, medRxiv, and Ovid. The search strategy was developed to capture the intersection of XAI, health care, and dentistry. The core query used was:

("explainable ai" or "interpretable machine learning" or "XAI") and ("healthcare" or "medical" or "clinical" or "diagnosis") and (dentistry) and (dental science)

The full search strategy for each database is provided in Supplementary File 2. Searches were executed on August 30, 2025. The search period was restricted to publications from 2015 onwards to ensure the inclusion of contemporary AI and XAI methodologies relevant to current dental clinical practice.

### Study selection

The study selection process adhered to the PRISMA 2020 directives^11^ (Figure) and followed a 4-stage process: identification, screening, eligibility, and inclusion. After deduplication, the titles and abstracts were independently screened by 2 reviewers (S.T. and V.T.) against the eligibility criteria. Full-text articles of potentially eligible studies were then assessed for inclusion. Any disagreements were resolved through discussion, and when consensus could not be reached, a third reviewer (T.P.) was consulted for final adjudication.

**Fig:**
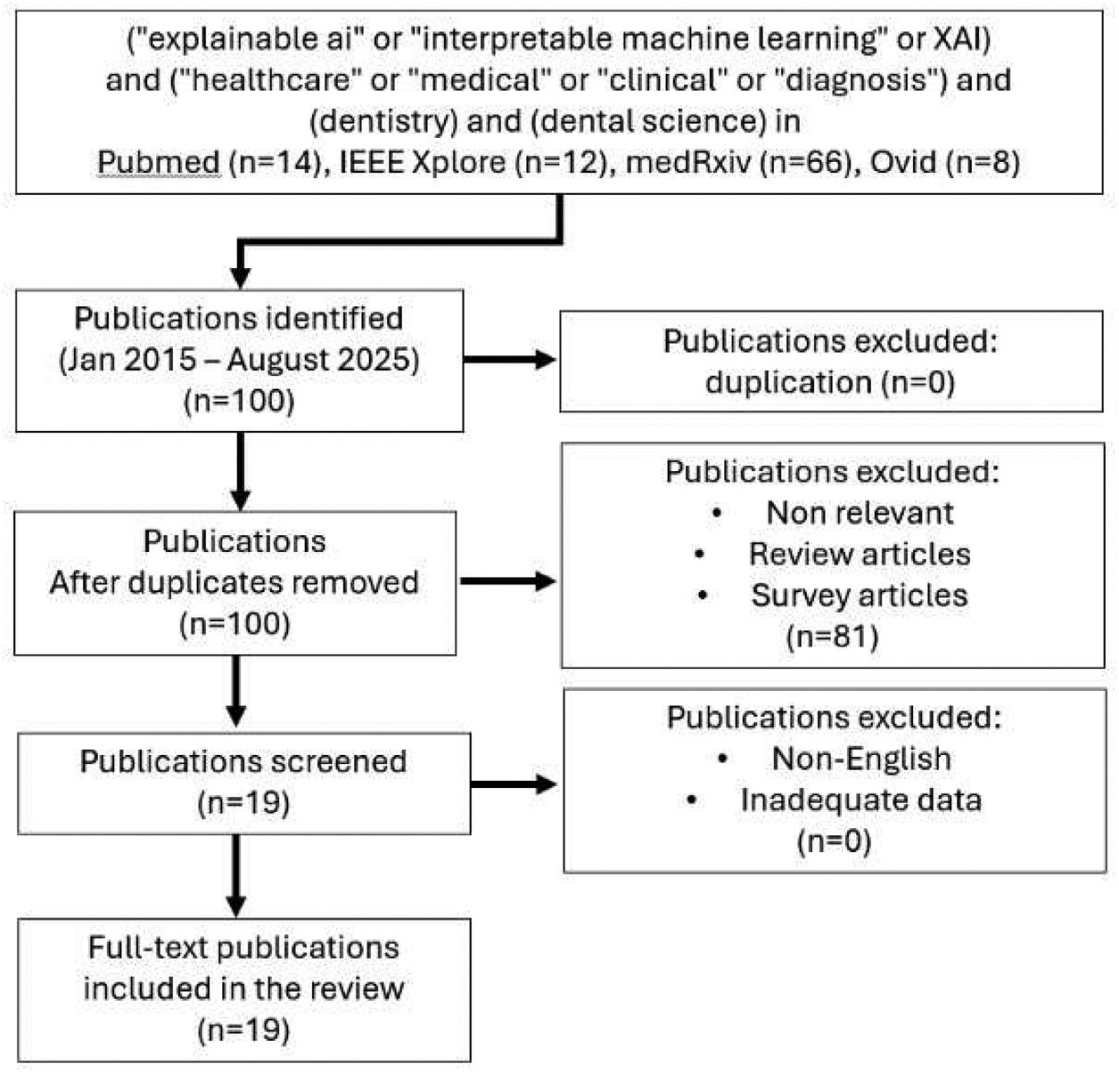
Preferred Reporting Items for Systematic Reviews and Meta-Analyses (PRISMA) 2020 flow diagram of the literature search and screening process. This diagram illustrates the flow of records through the systematic review process in accordance with PRISMA 2020. A total of 100 records were identified from PubMed (n = 14), IEEE Xplore (n = 12), medRxiv (n = 66), and Ovid (n = 8). After screening and eligibility assessment, 6 review articles and 1 survey study were excluded from PubMed, 6 studies without relevant keywords and 1 review article were excluded from IEEE Xplore, 59 manuscripts lacking XAI-related terms or proxy terms were excluded from medRxiv, and 5 studies without relevant keywords and 3 review articles were excluded from Ovid. Nineteen manuscripts were included in the final synthesis. XAI, explainable artificial intelligence.

### Data extraction

Data extraction was independently performed by 2 reviewers (S.T. and V.T.) using a predefined extraction framework, with disagreements resolved by consensus or adjudication by a third reviewer (T.P.) when necessary. To ensure alignment with methodological standards and the objectives of this review, the data extraction framework was developed by integrating PRISMA guidance with recommendations from recent reviews in explainable/dental AI research.^18^^,^^20^, ^21^, ^22^

For each included study, the following information was systematically extracted as follows:

1. *Bibliographic information*: author(s), year of publication, country of origin, and publishing journal or preprint platform
2. *Study characteristics*: study design (eg, retrospective, prospective, cross-sectional, experimental), sample size, and dataset type
3. *Clinical domain*: the clinical field addressed (eg radiology, oral pathology, orthodontics)
4. *AI model*: the machine learning or deep learning architecture employed (eg, convolutional neural network [CNN], transformer, ensemble methods, support vector machine)
5. *Explainable AI methodology*: the XAI techniques applied, including saliency maps (eg, Grad-CAM, heatmaps), feature attribution approaches (eg, SHAP, LIME), rule-based interpretable models, or other transparency-enhancing methods
6. *Evaluation metrics*: the performance outcomes reported, such as accuracy, area under the curve (AUC), sensitivity, specificity, F1-score, or interpretability and usability measures
7. *Clinical relevance*: the reported role of XAI within the dental or clinical context, including contributions to diagnosis, treatment planning, clinical decision support, or patient communication
8. *Study limitations*: author-identified challenges such as dataset size, validation strategy, generalisability, or interpretability constraints

The extraction framework was developed to ensure that the collected data were pertinent to the review’s primary objectives.

### Quality assessment

The methodological quality and risk of bias of included studies were critically appraised using tailored criteria from QUADAS-2^14^ for diagnostic accuracy studies and PROBAST^23^ for prediction model studies. Studies were evaluated across key domains: participant selection, predictor and outcome definition, analysis, and overall risk of bias. This assessment was central to our synthesis, moving beyond performance metrics to evaluate the foundational trustworthiness of the evidence.

## Results

### Study selection and characteristics

The PRISMA flow diagram (Figure) illustrates the screening process. From 100 initial records, 19 studies met the inclusion criteria. The included studies,^24-42^ summarised in Table 1, spanned a wide range of dental specialties, including cariology, periodontology, oral pathology, orthodontics, and forensic dentistry. The excluded studies, along with the reasons for exclusion, are summarised in Supplementary Table 1.

**Table 1:** Summary of included studies with condensed extraction (n = 19).

Bibliographic information	Study characteristics	Dental domain	AI model	XAI method	Evaluation metrics	Clinical relevance
Adnan et al^24^	Validation study; 7465 intraoral photos, Aga Khan University	Cariology	YOLOv5s CNN, DeTR	EigenCAM	Precision 90.7%, sensitivity 85.6%, F1 88.0%	Smartphone app detects caries comparably to junior dentists; scalable tool for low-resource settings
Dai et al^27^	Longitudinal; VicGen cohort (134 children), bariatric cohort (120 adults)	Paediatric dentistry	LP-Micro (ensemble RF, XGBoost, SVM, NN, LSTM, GRU, CNN-GRU)	Permutation importance (PermFit)	Improved vs baselines; *Streptococcus mutans* key ECC predictor	Highlighted microbial biomarkers for ECC prevention
Ikeda et al^38^	Prospective longitudinal; 61,883 older adults, JAGES study	Oral health/geriatric dentistry	RF, XGBoost, logistic regression	SHAP	Accuracy 0.88, F1 0.89, AUC 0.88	Identified oral health factors (teeth number, dry mouth) as fall predictors
Dai et al^28^	Longitudinal microbiome (VicGen, bariatric cohorts)	Paediatric dentistry	Lasso, XGBoost, RF, SVM, NN (LSTM, GRU, CNN-GRU)	Polynomial group lasso, permutation importance	AUC up to 0.74, accuracy 70%	Critical taxa/timepoints for ECC prediction
Parola et al^34^	Experimental; 567 oral lesion images	Oral cancer	YOLOv8, DETR, Faster R-CNN, ResNet, ensembles	Informed DL, case-based reasoning	mAP@50=0.732, accuracy 85.4% vs 77.7%	Explainable oral cancer screening robust to noisy images
Farook et al^39^	In vitro; 56 cavity preps (mandibular molars), ICDAS 3-6	Restorative dentistry	3D-CNN	Saliency maps	Accuracy 94.4%, DSC >0.90	3D scans classified cavity depth reliably
Kayadibi et al^31^	Retrospective; 1317 panoramic radiographs (UESB dataset)	Radiology (third molar detection)	CNN (E-mTMCNN, GoogLeNet, ResNet18, MobileNetV2, etc)	LIME	Accuracy 87%, AUC 0.87	Accurate third molar detection for treatment planning
Dangsungnoen et al^40^	User study, 24 participants (dental + data science students)	Forensic dentistry (sex prediction)	DeepToothDuo (EfficientNetB0), YOLOv5	OPG-SHAP + Gemini RAG explanations	Improved understanding (7.29), trust (3.85)	Dual-modality XAI enhanced comprehension/trust in sex prediction
Angelone et al^37^	Prospective; 79 patients, intraoral photos (plaque ROIs)	Periodontology	RF, NB, SVM, KNN, NNET, CART, LDA	Partial dependence plots	Accuracy up to 80%, specificity 100%	Explainable classification of moderate vs severe periodontitis
Kamran et al^25^	Dataset: 1593 periapical/panoramic x-rays	Cariology /hypodontia	Vision Transformer (ViT)	LIME, SHAP, Grad-CAM	Accuracy 96.6%, F1 96.6%	High-performance caries/hypodontia detection with interpretability
Milani et al^32^	CBCT (SOS staging), lateral cephalometric (malocclusion)	Orthodontics	ResNet18/34/50, ConvNeXt (+Attn)	Gradient attention maps, Grad-CAM	Accuracy up to 90.9%, AUC 93.5%	Verification of model attention alignment for orthodontics
Taskin and Al Islam et al^35^	Dataset: 12,320 images (6 oral disease classes)	General dentistry	MobileNetV2, DenseNet169, ResNet50V2, InceptionV3	Grad-CAM, LIME	Accuracy 89.0%, F1 89.1%	Robust interpretable multiclass oral disease diagnosis
Zhu et al^41^	NHANES data, >25,000 adults, nonradiographic multimodal	Periodontology	LR, RF, HGBT, SVM, 3-layer MLP	Feature importance, misclassification analysis	AUC 0.81, F1 0.74	Home-based periodontitis screening tool
Rivera et al^42^	Secondary dataset; 233 composites, 17 attributes, 7 outcomes	Dental materials	KNN, DT, RF, LR, SVM, XGBoost, Voting Regressor	Feature importance	Accuracy up to 0.90, ROC AUC 0.97, *R*² 0.93	Supports optimisation of restorative composite design
Pham^29^	58 paediatric panoramic radiographs	Paediatric dentistry	VLM (CNN + transformer)	Auto-generated text + saliency features	Accuracy 90%, AUC 0.96	Feasible multimodal diagnosis with VLMs
Pham^30^	70 train + 29 test paediatric panoramic radiographs	Paediatric dentistry	1D-CNN, LSTM, BERT; pretrained CNNs	Proxy XAI: text descriptions via ChatGPT	1D-CNN accuracy 84%, BERT 76.7%, LSTM 56.7%	Text-based classification improved interpretability
Motmaen et al^36^	Retrospective; 1,184 PANs (26,956 teeth)	Oral surgery	ResNet-50	CAMERAS (activation mapping)	ROC-AUC 0.901, PR-AUC 0.749	AI > dentists for extraction prediction
Devlin et al^26^	RCT; 23 dentists, 24 bitewing radiographs	Cariology /radiology	AssistDent ML prompts	On-screen saliency prompts	Sensitivity 75.8% vs 44.3%, specificity 85.4% vs 96.3%	AI significantly improved enamel-only caries detection
Lee et al^33^	Retrospective; 151 cephalometric cases	Oral and maxillofacial surgery	LLMs (LLAMA-2, GPT-3.5/4, Gemini-Pro)	Prompt engineering, structured text conversion	Balanced accuracy up to 67.7%, F1 up to 67.5%	LLMs feasible for cephalometric diagnosis

Abbreviations: 1D, 1-dimensional; 3D, 3-dimensional; AI, artificial intelligence; AUC, area under the curve; BERT, bidirectional encoder representations from transformer; CART, classification and regression trees; CBCT, cone-beam computed tomography; CNN, convolutional neural network; DETR, detection transformer; DL, deep learning; DSC, dice similarity coefficient; DT, decision tree; ECC, early childhood caries; E-mTMCNN, explainable mandibular third molar convolutional neural network; GRU, gated recurrent unit; HGBT, histogram-based gradient boosting tree; ICDAS, international caries detection and assessment system; KNN, k-nearest neighbor; LDA, linear discriminant analysis; LIME, local interpretable model-agnostic explanations; LLM, large language model; LP-Micro, longitudinal microbiome-based interpretable machine-learning framework; LR, learning rates; LSTM, long short-term memory; ML, machine learning; MLP, multilayer perceptron; NB, naive bayes; NHANES, National Health and Nutrition Examination Survey; NN, neural networks; NNET, neural networks; OPG, orthopantomogram; PAN, panoramic radiograph; PR-AUC, panoramic radiography area under the curve; RAG, retrieval-augmented generation; R-CNN, region-based convolutional neural network; RCT, randomized controlled trial; RF, random forest; ROC, receiver operating characteristic; ROI, region of interest; SHAP, shapley additive explanations; SOS, spheno-occipital synchondrosis; SVM, support vector machine; UESB, universidade estadual do sudoeste da bahia; VLM, vision language model; XAI, explainable artificial intelligence; XGBoost, extreme gradient boosting.

CNNs were the predominant AI architecture. The most common XAI techniques were Grad-CAM, SHAP, and LIME.

The main study characteristics are summarised in Table 1, while complete details, including dataset properties, performance metrics, and XAI methods, are available in Supplementary Table 2.

### Cariology

In cariology, several studies focused on caries detection from radiographs or photographs. Adnan et al^24^ validated YOLOv5s- and DeTR-based detectors of 7465 intraoral images, demonstrating high precision (90.7%) and an F1-score (88.0%) for caries detection via a smartphone application. Kamran et al^25^ used Vision Transformer models with SHAP, LIME, and Grad-CAM to detect caries and hypodontia from periapical and panoramic radiographs, achieving 96.6% accuracy. Devlin et al^26^ conducted a randomised controlled trial using AssistDent, reporting a marked increase in sensitivity for detecting enamel-only caries compared to dentists without AI support.

### Paediatric dentistry

In paediatric dentistry, Dai et al^27^^,^^28^ used ensemble machine learning and longitudinal microbiome data from the VicGen and bariatric cohorts to identify microbial biomarkers predictive of early childhood caries. Pham^29^^,^^30^ investigated paediatric panoramic radiographs using multimodal and text-based methods. A vision-language model combining CNN and transformer layers achieved 90% accuracy and an AUC of 0.96, while proxy-based XAI models using text descriptions demonstrated that a 1-dimensional CNN outperformed pretrained CNNs and a pretrained bidirectional transformer language model (BERT) in interpretability for binary disease classification.

### Radiology and imaging

Radiology and imaging studies were also prominent. Kayadibi et al^31^ trained CNN architectures (eg, ResNet18, MobileNetV2) of 1317 panoramic radiographs for third molar detection, applying LIME to provide feature explanations and achieving 87% accuracy. Milani et al^32^ applied ResNet and ConvNeXt models to cephalometric and cone-beam computed tomography (CBCT) datasets, integrating attention maps and Grad-CAM to verify orthodontic staging with an AUC of up to 93.5%. Lee et al^33^ explored the feasibility of large language models (LLMs), including GPT-4 and Gemini-Pro, for cephalometric diagnosis, reporting balanced accuracies of 67.7%.

### Oral pathology and cancer detection

In oral pathology and cancer detection, Parola et al^34^ evaluated YOLOv8, DETR, and Faster R-CNN models with case-based reasoning for oral lesion recognition, achieving a mAP@50 of 0.732. Taskin and Al Islam^35^ developed an interpretable multiclass model (MobileNetV2, DenseNet, ResNet, Inception) to classify 6 oral diseases with 89% accuracy. Motmaen et al^36^ trained a ResNet-50 on 26,956 teeth from 1184 panoramic radiographs to predict tooth extraction decisions; their model, which used CAMERAS for activation mapping, outperformed dentists (receiver operating characteristic AUC = 0.901).

### Other dental specialties

Other dental specialties were also included in our review. Angelone et al^37^ applied interpretable machine learning (ML) models, with partial dependence plots, to intraoral plaque biofilm images, achieving 80% accuracy for classifying periodontal disease severity. Ikeda et al^38^ leveraged SHAP to explain risk factors for falls among 61,883 older adults, demonstrating the clinical utility of oral health data in geriatric dentistry. Farook et al^39^ developed a 3-dimensional CNN with saliency maps to classify cavity depth in restorative dentistry, achieving 94.4% accuracy. Dangsungnoen et al^40^ proposed DeepToothDuo with SHAP and retrieval-augmented explanations for forensic sex prediction, which improved participant trust and understanding. Other reviewed studies included those of Zhu et al,^41^ who trained multimodal ML models on National Health and Nutrition Examination Survey data to predict periodontitis with an AUC of 0.81, suggesting a pathway to home-based screening, and Rivera et al,^42^ who examined dental composite performance using feature importance from multiple ML models to support material optimisation.

Together, these studies demonstrate the diversity of XAI applications in dentistry, spanning diagnostic imaging, preventive care, restorative materials, and clinical decision support.

## Synthesis of AI and XAI methodological trends

A clear pattern has emerged in the pairing of XAI techniques with AI models and data types. Saliency-based methods (Grad-CAM, heatmaps) were almost exclusively applied to CNN-based image analysis.^24^^,^^25^^,^^31^^,^^32^^,^^35^^,^^36^^,^^43^ In contrast, feature attribution methods like SHAP were typically used with tree-based models or tabular data.^27^^,^^28^^,^^38^^,^^41^^,^^42^ This reflects a technically appropriate but largely formulaic application of XAI. Only 1 study by Dangsungnoen et al^40^ explicitly designed and evaluated a multimodal explanation system (OPG-SPELL) with end users, measuring its impact on understanding and trust.

## Critical appraisal of methodological rigour and risk of bias

The quality assessment of the evaluated studies reveals a field in its infancy, grappling with fundamental methodological challenges. Most studies (14 of 19) were judged to have a high overall risk of bias as discussed below.

The methodological quality of the 19 included studies was appraised using QUADAS-2 for diagnostic accuracy and PROBAST for prediction model studies. Across the body of evidence, the risks of bias and applicability concerns varied considerably depending on study design, population, and reference standards. The domain-level assessments are summarised in Table 2, with complete details available in Supplementary Table 3.

**Table 2:** Quality assessment of included studies (n = 19).

Bibliographic information	QUADAS-2*					PROBAST†				Overall risk of bias‡
	Patient selection	Index test	Reference standard	Flow and timing	Applicability concerns	Participants	Predictors	Outcomes	Analysis	
Adnan et al^24^	High	Low	High	Low	High	High	Low	High	High	High
Dai et al^27^	Low	Low	Low	Low	Fair	High	Low	Low	High	Fair
Ikeda et al^38^	Low	Low	Low	Low	Low	Low	Low	High	Low	Fair
Dai et al^28^	Low	Low	Low	Low	Low	Low	Low	Low	Fair	Low
Parola et al^34^	High	Fair	High	Fair	High	High	Fair	Fair	High	High
Farook et al^39^	High	Low	Low	Low	High	High	Low	Low	High	High
Kayadibi et al^31^	Low	Low	Low	Limited	Low	High	Low	Low	High	Fair
Dangsungnoen et al^40^	High	Low	High	Low	High	High	Low	High	High	High
Angelone et al^37^	High	Low	High	Low	Fair	High	Low	High	High	High
Kamran et al^25^	High	Low	High	High	High	High	Low	High	High	High
Milani et al^32^	High	Low	Fair	Limited	Fair	High	Low	Fair	Fair	Fair
Taskin and Al Islam et al^35^	High	Low	High	Limited	High	High	Low	Fair	High	High
Zhu et al^41^	Low	Low	High	High	Low	Low	Low	High	Low	Fair
Rivera et al^42^	High	Low	High	High	High	High	Low	Low	High	High
Pham^29^	High	Low	High	Limited	Fair	High	Low	Fair	High	High
Pham^30^	High	Low	Low	High	High	High	Low	Low	High	High
Motmaen et al^36^	High	Low	Fair	Low	Fair	High	Low	Fair	High	High
Devlin et al^26^	Low	Low	Low	Low	Low	Low	Low	Low	Low	Low
Lee et al^33^	High	High	High	High	Limited	High	High	High	High	High

⁎ For all QUADAS-2 assessment. “Low” means low risk of bias with clearly defined methodology and appropriate reference standards; “Fair” means some methodological concerns with potential bias but not likely to alter results substantially; “High” means high risk of bias due to major flaws such as limited sample size, weak reference standards, or lack of external validation; and “Limited” means insufficient information reported to permit a clear judgement.

† For all PROBAST assessment. “Low” indicates low risk of bias with adequately described participants, predictors, outcomes, and analyses; “Fair” indicates some concerns, generally robust but with restricted generalisability; and “High” indicates high risk of bias due to methodological shortcomings, poor representativeness, or risk of overfitting.

‡ For overall risk of bias assessment. “Low” means low concern across all domains; “Fair” means some concerns that may limit generalisability but unlikely to invalidate findings; “High” means substantial concerns likely to compromise validity.

Several studies were judged to have a high overall risk of bias, primarily due to small, single-centre datasets and nonstandard outcome definitions. For instance, Adnan et al^24^ and Parola et al^34^ relied on convenience samples and nonexpert or imperfect reference standards, raising concerns in the outcome domains of both QUADAS-2 and PROBAST. Similarly, Farook et al^39^ and Pham^29^^,^^30^ employed very small in vitro or paediatric datasets, which limited their external validity. Studies by Kamran et al,^25^ Taskin and Al Islam,^35^ Rivera et al,^42^ Motmaen et al,^36^ and Lee et al^33^ also showed high risks, stemming from their reliance on public or retrospective datasets, a lack of external validation, or insufficiently representative participant populations.

In contrast, a subset of studies demonstrated low to moderate risks of bias, often supported by larger or more representative cohorts. Dai et al^27^^,^^28^ achieved low-risk ratings across most QUADAS-2 and PROBAST domains by employing prospective or multicohort microbiome datasets with well-defined predictors and outcomes, although concerns remained regarding limited generalisability. Ikeda et al^38^ also found low risk in most domains, although the use of self-reported outcomes introduced moderate concerns. Zhu et al^41^ leveraged a large, nationally representative dataset (National Health and Nutrition Examination Survey) that was rated low risk in participant and predictor domains; however, its outcome definitions did not align with gold-standard periodontal diagnoses.

Other studies presented methodologically balanced designs but were limited in scope. Kayadibi et al^31^ and Milani et al^32^ featured clearly defined predictors and robust internal validation but were confined to single-institution data and lacked external testing. Similarly, Angelone et al^37^ and Dangsungnoen et al^40^ showed innovative applications of XAI but were hampered by small sample sizes and nonstandard outcome measures. Conversely, the ADEPT study^26^ achieved an overall low risk of bias, benefitting from its randomised design, expert gold-standard comparators, and consistent participant assessment, despite a modest sample size.

Overall, most studies were hampered by concerns regarding external validity, dataset representativeness, and a lack of standardised reference standards. While a minority demonstrated robust methodological practices with low risks of bias, most presented high risks in at least 1 domain, particularly in outcome assessment and external validation.

*Patient selection*: A predominant issue was the use of small, retrospective, single-centre datasets, often from public repositories, leading to high concerns about representativeness and generalisability.

*Reference standard and outcomes*: Many studies used nonexpert annotations or imperfect reference standards, casting doubt on the validity of the “ground truth” used to train and evaluate the models.

*Analysis*: A near-universal limitation was the absence of external validation. Models were typically trained and tested on data from the same source, creating a high risk of overfitting and severely limiting claims of generalisability. Only a single study, the ADEPT randomised controlled trial,^26^ was rated as having a low risk of bias across all domains, demonstrating the feasibility of robust study design in this field.

## Discussion

This systematic review synthesises the current landscape of XAI in dentistry and delivers a critical message: while the technical application of XAI is growing, the evidence base for its ability to foster genuine clinical trust remains weak. Our analysis, anchored by a formal quality assessment, identifies several critical themes that must be addressed for the field to advance.

In general, we submit that there was a deficit of methodological rigour in most of the 19 studies reviewed. CNNs were the most frequently adopted architecture, with interpretability commonly enabled through saliency-based visualisation tools such as Grad-CAM and heatmaps.^43^^,^^44^ Other approaches, including model-agnostic techniques such as SHAP and LIME and attention mechanisms in transformer-based models, have also been reported to support interpretability of predictions derived from both imaging and structured data.

By appraising these studies, this review demonstrates that while XAI has begun to permeate dental AI research, its implementation is uneven and frequently limited to proof-of-concept applications. Some investigations provided robust interpretability frameworks that aligned computational outputs with clinical reasoning, thereby supporting clinician trust in diagnostic decisions.^26^, ^27^, ^28^^,^^38^^,^^41^ Others offered only partial or rudimentary explanations, raising concerns about transparency and reproducibility.^24^^,^^25^^,^^29^, ^30^, ^31^, ^32^, ^33^, ^34^, ^35^, ^36^, ^37^^,^^39^^,^^40^^,^^42^

Synthesising these findings allows us to assess not only the extent of XAI adoption but also the degree to which current evidence addresses the core challenge of trustworthiness in dental AI. In doing so, the review fulfils its objective of identifying, appraising, and critically evaluating XAI applications in dentistry to determine whether explainability meaningfully enhances trust in diagnostic decision-making.

The most significant finding of this review is the pervasive high risk of bias in the included studies. This has a direct and profound implication for trustworthiness. An explanation, no matter how visually compelling or intuitively appealing, is meaningless if it is explaining the behaviour of a flawed or nongeneralisable model. For instance, a Grad-CAM heatmap highlighting a specific region on a radiograph provides a false sense of security if the underlying model was trained on a small, biased dataset and never validated externally. The dental XAI community must first conquer these foundational challenges of dataset quality, robust validation, and outcome design before the nuanced benefits of explainability can be realised. Trust in clinical AI is built on proven reliability, for which transparency is a complement, not a substitute.

### The chasm between technical and clinical explainability

There is a stark disconnect between the technical implementation of XAI and its clinical evaluation. The literature is dominated by post hoc explanations applied to black box models, with the validity of the explanation often assumed rather than tested. Very few studies ventured beyond technical validation to ask: Does this explanation actually help a dentist?

Does a SHAP value or a LIME plot improve diagnostic confidence? Does it reduce error rates? Does it facilitate communication with a patient? The study by Dangsungnoen et al^40^ is a notable exception, demonstrating that designed explanations can improve user understanding. The field must shift from demonstrating that an AI model can be explained to proving that the explanation is useful in a clinical workflow. This requires a new paradigm of human-centred design and evaluation, involving dental professionals as end-users throughout the development process.

### The unexplored potential of inherently interpretable models

The current dental XAI landscape is overwhelmingly focused on explaining complex models (“post hoc explainability”). The debate in the wider XAI literature, however, questions whether this approach can ever be fully trustworthy.^45^ An alternative path is to use inherently interpretable models (eg, decision trees, linear models, rule-based systems) that are transparent by design. While these may sometimes sacrifice marginal performance, their decisions are inherently understandable. With the exception of a few studies using models such as random forests or logistic regression with SHAP,^37^^,^^38^^,^^41^ this approach is underexplored in dentistry. For many clinical tasks, the high-stakes nature of decision-making may favour the certifiable trust of an interpretable model over the uncertain explanation of a more complex one.

The findings of this review suggest that while XAI methods are increasingly incorporated into dental diagnostic research, their readiness for clinical adoption remains limited. Across the 19 included studies, saliency-based methods such as Grad-CAM and heatmaps were the most frequently applied, often serving to visually justify CNN predictions. Other studies adopted feature attribution techniques, such as SHAP or LIME, or attention-based mechanisms embedded within transformers. These strategies provide an important first step towards transparency, offering clinicians insight into why predictions are made. However, the evidence presented remains largely experimental: most studies relied on retrospective datasets with limited diversity and modest sample sizes, and only a minority reported external validation. Equally important, few studies explicitly evaluated clinician-centred outcomes, such as whether XAI explanations improved diagnostic confidence, reduced error rates, or enhanced communication with patients. Without prospective, real-world testing and without clear demonstration that interpretability meaningfully improves decision-making or workflow efficiency, the ability of current XAI implementations to enhance trust and support routine adoption remains uncertain.

In the wider medical literature, several reviews have directly addressed barriers to the clinical adoption of XAI. Tonekaboni et al^19^ emphasised that interpretability must be evaluated in terms of its impact on clinical decision-making and patient outcomes rather than technical plausibility alone. Amann et al^17^ highlighted regulatory, ethical, and usability challenges that hinder XAI integration into health care workflows, while Rudin et al^46^ argued that inherently interpretable models may offer stronger trust foundations than post hoc explanation tools. Compared to these health care–focused reviews, the current dental XAI landscape remains at an earlier stage: none of the dental studies included incorporated prospective clinical validation, usability testing with practitioners, or patient-centred measures of interpretability. This gap underscores the need for future dental research to move beyond proof-of-concept demonstrations and adopt evaluation frameworks that explicitly measure whether XAI explanations improve clinical adoption and trust in practice.

### Limitations of the review

Our review has a few limitations. The restriction to English-language studies and the specific databases searched may have omitted relevant work. The rapid evolution of AI means that some very recent advances may not be captured. Furthermore, the application of QUADAS-2 and PROBAST, while appropriate, requires adaptation for some AI-specific studies, which may introduce subjectivity.

## Future directions

This review demonstrates that the application of XAI in dentistry is widespread but shallow. The field is currently characterised by technically focused proofs of concept that are built upon methodologically weak foundations. To transition from a promising concept to a trusted clinical tool, we propose a concerted shift in research priorities such as universal adoption of standardised reporting measures, prospective validation, and real-world integration, focusing on trust, reproducibility, and clinically grounded evaluation.

*Prioritising robustness*: Future studies must adhere to higher methodological standards. This includes using larger, prospectively collected, and diverse datasets; implementing rigorous external validation; and employing clinically accepted reference standards. The reporting of studies should be standardised to include key details on data provenance, model selection, and hyperparameter.

*Development of standardised metrics*: Research organisations such as the International Association for Dental, Oral, and Craniofacial Research (IADR) and nongovernmental organisations akin to the International Dental Federation must, in partnership, develop and adopt standardised metrics and frameworks for evaluating XAI’s impact on human users. This involves moving beyond accuracy and AUC to measure outcomes such as diagnostic confidence, time to decision, error detection, and user satisfaction through controlled studies with dentists.

*Human-centred design*: XAI systems should be codesigned with clinicians to ensure that the explanations provided are relevant, intuitive, and integrated into the clinical workflow. Research should explore what information dentists need, in what format, and at what point in the decision-making process. Human-centred evaluation remains underexplored in current XAI research. Future studies should incorporate structured comparisons between explainable AI systems and human examiners to better contextualise model explanations, clinical trust, and decision-making relevance.

*Reconsidering the model paradigm*: Researchers should critically evaluate whether a complex, post hoc explained model is always necessary. For many tasks, inherently interpretable models may provide a more direct and trustworthy path to clinical adoption. In other words, very often a simpler, easier-to-understand model is the smarter and more trustworthy choice for a clinical setting.

## Conclusion

In conclusion, the journey toward trustworthy AI in dentistry is a marathon, not a sprint. The widespread but often superficial application of XAI techniques is the first step. The next, more challenging step is to build these techniques upon an unshakeable foundation of methodological rigour and to prove their value not just in silico, but in the hands of the clinicians they are meant to serve.

## Author contributions

S.T., V.T.: investigation, data analysis, and drafting the manuscript. H.A.R, L.S., and T.O.: data analysis and edited the manuscript. T.P.: study conception, data acquisition and interpretation, drafted the manuscript. All authors critically revised the manuscript and gave their final approval for publication.

## Conflict of interest

The authors declared no potential conflicts of interest with respect to the research, authorship, and/or publication of this article.

## Data availability statement

Original data generated and analysed during this study are included in this published article or in the supplementary material.

## Appendix Supplementary materials

Supplementary File 1: PRISMA 2020 checklist Image, application 1

Supplementary File 2: Database search strategy Image, application 2

Supplementary Table 1: Full-text articles excluded after eligibility assessment and reasons for exclusion. Image, application 3

Supplementary Table 2: Detailed data extraction of included studies. Image, application 4

Supplementary Table 3: Detailed explanations for quality assessment of included studies. Image, application 5

## Footnotes

Supplementary material associated with this article can be found in the online version at doi:10.1016/j.identj.2026.109626.
