# Supplementary material for: Explainable Artificial Intelligence in Dentistry: A Systematic Review of Its Trust and Translation
Source: Int Dent J. 2026 May 25;76(4):109626. doi: 10.1016/j.identj.2026.109626 (PMC13223827; doi:10.1016/j.identj.2026.109626)
Supplement: Supplementary file 2 — Database search strategy [file mmc2.docx]

**Database search strategy**

**The search strategy was as follows:**

- **PubMed:** ("explainable ai" OR "interpretable machine learning" OR xai) AND (healthcare OR medical OR clinical OR diagnosis) AND (dentistry) AND (dental science)
- **IEEE Xplore:** ("All Metadata":"explainable ai" OR "All Metadata":"interpretable machine learning" OR "All Metadata":xai) AND ("All Metadata":healthcare OR "All Metadata":medical OR "All Metadata":clinical OR "All Metadata":diagnosis) AND ("All Metadata":dentistry OR "All Metadata":"dental science")
- **medRxiv (Dentistry and Oral Medicine category):** explainable ai OR interpretable machine learning OR xai
- **Ovid:** (FullText:(AllFields:("explainable ai" OR "interpretable machine learning" OR "xai") AND ("healthcare" OR "medical" OR "clinical" OR "diagnosis") AND (dentistry) AND (dental science)))

The searches were carried out on 30 August 2025. To maintain relevance to contemporary AI methodologies, we restricted our review to studies published between January 2015 and August 2025.
