# Supplementary material for: Explainable Artificial Intelligence in Dentistry: A Systematic Review of Its Trust and Translation
Source: Int Dent J. 2026 May 25;76(4):109626. doi: 10.1016/j.identj.2026.109626 (PMC13223827; doi:10.1016/j.identj.2026.109626)
Supplement: Supplementary file 3 — Supplementary Table 1: Full-text articles excluded after eligibility assessment and reasons for exclusion. [file mmc3.docx]

**Supplementary Table 1. full-text articles excluded after eligibility assessment and reasons for exclusion.**

| **PMID/DOI/ISBN** | **Title** | **Database** | **Reason for Exclusion** |
| --- | --- | --- | --- |
| 33340123 | Promises and perils of artificial intelligence in dentistry | PubMed | Not an original research article |
| 37639191 | New trend in artificial intelligence-based assistive technology for thoracic imaging | PubMed | Not an original research article |
| 39010644 | Deep learning and explainable artificial intelligence for investigating dental | PubMed | Not an original research article |
| 39092200 | Responsible artificial intelligence for addressing equity in oral healthcare | PubMed | Not an original research article |
| 39266401 | Artificial Intelligence-Related Dental Research: Bibliometric and Altmetric Analysis | PubMed | Not an original research article |
| 39969623 | Exploring a decade of deep learning in dentistry: A comprehensive mapping review | PubMed | Not an original research article |
| 40863069 | Charting New Territory: AI Applications in Dental Caries Detection from Panoramic Imaging | PubMed | Not an original research article |
| 10.1109/ISCV60512.2024.10620154 | Process Quality Assurance of Artificial Intelligence in Medical Diagnosis | IEEE Xplore | Not related to dentistry/oral health |
| 10.1109/SysCon61195.2024.10553620 | Systems Modeling of Trust in AI-Enabled Medical Diagnosis | IEEE Xplore | Not related to dentistry/oral health |
| 10.1109/EMBC53108.2024.10781771 | An Explainable and Conformal AI Model to Detect Temporomandibular Joint Involvement in Children Suffering from Juvenile Idiopathic Arthritis | IEEE Xplore | Not related to dentistry/oral health |
| 9788770228848 | 10 Explainable Artificial Intelligence Applications in Dentistry: A Theoretical Research | IEEE Xplore | Not an original research article |
| 10.1109/DASA63652.2024.10836302 | Classification of Lung Diseases Using Machine Learning Technique | IEEE Xplore | Not related to dentistry/oral health |
| 10.1109/BMEiCON64021.2024.10896299 | Booklet | IEEE Xplore | No explicit explainable AI methodology |
| 10.1109/FUZZ-IEEE60900.2024.10611999 | A Type-2 Fuzzy Logic-Based Explainable Artificial Intelligence for the Prediction of Enhancers | IEEE Xplore | Not related to dentistry/oral health |
| N/A (preprint) | Multi-level Regulatory Roles of Lactate Metabolism Gene Network in Oral Cancer: Machine Learning Insights | medRxiv | No explicit explainable AI methodology) |
| N/A (preprint) | Pan-Omics Fusion and Machine Learning Unveil Congenital Tooth Agenesis–Ecto-mesodermal Diseases Link and Biomarker Discovery | medRxiv | No explicit explainable AI methodology |
| N/A (preprint) | Routine Blood Tests as Predictors of Periodontal Disease Risk Using a Machine Learning Approach | medRxiv | No explicit explainable AI methodology |
| N/A | Deep learning-based localization of bounded edentulous spaces in intraoral occlusal images | medRxiv | No explicit explainable AI methodology |
| N/A | Classification of Pediatric Dental Diseases from Panoramic Radiographs using Natural Language Transformer and Deep Learning Models | medRxiv | No explicit explainable AI methodology |
| N/A | Integrating Support Vector Machines and Deep Learning Features for Oral Cancer Histopathology Analysis | medRxiv | No explicit explainable AI methodology |
| N/A | DentalSegmentator: robust deep learning-based CBCT image segmentation | medRxiv | No explicit explainable AI methodology |
| N/A | Deep Learning Approach to Measure Alveolar Bone Loss After COVID-19 | medRxiv | No explicit explainable AI methodology |
| N/A | A Novel Collaborative Learning Model for Teeth and Fillings in Radiographs | medRxiv | No explicit explainable AI methodology |
| N/A | Automatic Three-Dimensional Cephalometric Landmarking via Deep Learning | medRxiv | No explicit explainable AI methodology |
| N/A | Application of deep learning to classify skeletal growth phase on 3D radiographs | medRxiv | No explicit explainable AI methodology |
| N/A | Deep Learning for Caries Detection using Optical Coherence Tomography | medRxiv | No explicit explainable AI methodology |
| N/A | The use of artificial intelligence in the diagnosis of carious lesions: Systematic review and meta-analysis | medRxiv | No explicit explainable AI methodology |
| N/A | A Pilot Comparative Study of Dental Students’ Ability to Detect Enamel-only Proximal Caries in Bitewing Radiographs With and Without the use of AssistDent® Deep Learning Software | medRxiv | No explicit explainable AI methodology |
| N/A | Development and validation of diagnostic and prognostic prediction tools for dental caries in young children: A protocol | medRxiv | Not an original research article |
| N/A | Comparison of binary classifiers in forensic dentistry for sex determination | medRxiv | No explicit explainable AI methodology |
| N/A | Informatic tools for diagnosis in dentistry. A compilation review | medRxiv | Not an original research article |
| N/A | OQA: A question-answering dataset on orthodontic literature | medRxiv | No explicit explainable AI methodology |
| N/A | Artificial Intelligence in Periodontology: Performance Evaluation of ChatGPT, Claude, and Gemini on the In-service Examination | medRxiv | No explicit explainable AI methodology |
| N/A | Deep convolution neural network for screening carotid calcification in dental panoramic radiographs | medRxiv | No explicit explainable AI methodology |
| N/A | SISTR: Sinus and Inferior alveolar nerve Segmentation with Targeted Refinement on Cone Beam Computed Tomography images | medRxiv | No explicit explainable AI methodology |
| N/A | Detecting salivary host-microbiome RNA signature for aiding diagnosis of oral and throat cancer | medRxiv | No explicit explainable AI methodology |
| N/A | Morphometric variations of human mandible in Indian population: comparison between subjects having healthy and ankylosed temporomandibular joint | medRxiv | No explicit explainable AI methodology |
| N/A | Trabeculae microstructure parameters serve as effective predictors for marginal bone loss of dental implant in the mandible | medRxiv | No explicit explainable AI methodology |
| N/A | International Expert-Based Consensus Definition, Staging Criteria, and Minimum Data Elements for Osteoradionecrosis of the Jaw: An Inter-Disciplinary Modified Delphi Study | medRxiv | No explicit explainable AI methodology |
| N/A | Exploring the use of preprints in dentistry | medRxiv | No explicit explainable AI methodology |
| N/A | Recovering Mandibular Morphology after Disease with Artificial Intelligence | medRxiv | No explicit explainable AI methodology |
| N/A | Salivary proteome of aphthous stomatitis reveals the participation of vitamin metabolism, nutrients, and bacteria | medRxiv | No explicit explainable AI methodology |
| N/A | Saliva as a Candidate for COVID-19 Diagnostic Testing: A Meta-Analysis | medRxiv | No explicit explainable AI methodology |
| N/A | BURNOUT: A PREDICTOR OF ORAL HEALTH IMPACT PROFILE AMONG NIGERIAN EARLY CAREER DOCTORS | medRxiv | No explicit explainable AI methodology |
| N/A | Artificial Intelligence in Periodontology: Performance Evaluation of ChatGPT, Claude, and Gemini on the In-service Examination | medRxiv | No explicit explainable AI methodology |
| N/A | Deep learning-based localization of bounded edentulous spaces in intraoral occlusal images | medRxiv | No explicit explainable AI methodology |
| N/A | Dental Composite Performance Prediction Using Artificial Intelligence | medRxiv | Not related to dentistry/oral health |
| N/A | Prevalence of Tobacco Pouch Keratosis in India: A Systematic Review and Meta-Analysis Protocol | medRxiv | Not an original research article |
| N/A | Informatic tools for diagnosis in dentistry. A compilation review | medRxiv | Not an original research article |
| N/A | Comparative Success and Survival of Preformed Metal Crown Treatment Between the Hall Technique and Conventional Technique: An Umbrella Review | medRxiv | Not an original research article |
| N/A | SISTR: Sinus and Inferior alveolar nerve Segmentation with Targeted Refinement on Cone Beam Computed Tomography images | medRxiv | No explicit explainable AI methodology |
| N/A | Integrating Support Vector Machines and Deep Learning Features for Oral Cancer Histopathology Analysis | medRxiv | No explicit explainable AI methodology |
| N/A | The use of artificial intelligence in the diagnosis of carious lesions: Systematic review and meta-analysis | medRxiv | Not an original research article |
| N/A | Association of Salivary Biomarker Concentration and Activity Between Caries-free and Caries-affected Children: An Umbrella Review | medRxiv | Not an original research article |
| N/A | Oral Health Research Across the Lifespan: A Systematic Mapping Review of Cohort Studies in Australia and New Zealand | medRxiv | Not an original research article |
| N/A | Development and validation of diagnostic and prognostic prediction tools for dental caries in young children: A protocol | medRxiv | No explicit explainable AI methodology |
| N/A | Assessing the impact of periodontal therapy on tooth loss: a register-based longitudinal study in Denmark | medRxiv | No explicit explainable AI methodology |
| N/A | Effect of plasma treatment on the shear bond strength of ceramic and composite to human dental enamel | medRxiv | No explicit explainable AI methodology |
| N/A | Deep Learning Approach to Measure Alveolar Bone Loss After COVID-19 | medRxiv | No explicit explainable AI methodology |
| N/A | Omega-3 & Aspirin Boost in Periodontal Therapy for Type II Diabetes: A Systematic Review | medRxiv | Not an original research article |
| N/A | Early Imaging Identification of Osteoradionecrosis and Classification Using the Novel ClinRad System | medRxiv | No explicit explainable AI methodology |
| N/A | OQA: A question-answering dataset on orthodontic literature | medRxiv | No explicit explainable AI methodology |
| N/A | The effect of enzyme and protein containing toothpaste on gingival condition: a randomised controlled study | medRxiv | No explicit explainable AI methodology |
| N/A | DentalSegmentator: robust deep learning-based CBCT image segmentation | medRxiv | No explicit explainable AI methodology |
| N/A | Features and Networks of the Mandible on Computed Tomography | medRxiv | No explicit explainable AI methodology |
| N/A | Personalized oral care (Precaries): an intervention study customized according to genetic cause and risk | medRxiv | No explicit explainable AI methodology |
| N/A | Morphological changes in the mandible associated with the presence of exostoses: a study in two archaeological populations from southern France | medRxiv | No explicit explainable AI methodology |
| N/A | Analysis of Dental Tissues Density in Healthy Children Based on Radiological Data | medRxiv | No explicit explainable AI methodology |
| N/A | Deep convolution neural network for screening carotid calcification in dental panoramic radiographs | medRxiv | No explicit explainable AI methodology |
| N/A | A Pilot Comparative Study of Dental Students’ Ability to Detect Enamel-only Proximal Caries in Bitewing Radiographs With and Without the use of AssistDent® Deep Learning Software | medRxiv | No explicit explainable AI methodology |
| N/A | Deep Learning for Caries Detection using Optical Coherence Tomography | medRxiv | No explicit explainable AI methodology |
| N/A | School-based caries prevention using silver diamine fluoride: A pragmatic randomized trial in low-income rural children | medRxiv | No explicit explainable AI methodology |
| N/A | Diagnosis of Pathological Speech with Efficient and Effective Features for Long Short-Term Memory Learning | medRxiv | No explicit explainable AI methodology |
| N/A | An open-label, parallel-group, randomized clinical trial of different silver diamine fluoride application intervals to arrest dental caries | medRxiv | No explicit explainable AI methodology |
| N/A | Application of deep learning to classify skeletal growth phase on 3D radiographs | medRxiv | No explicit explainable AI methodology |
| N/A | A Novel Collaborative Learning Model for Teeth and Fillings in Radiographs | medRxiv | No explicit explainable AI methodology |
| N/A | International Expert-Based Consensus Definition, Staging Criteria, and Minimum Data Elements for Osteoradionecrosis of the Jaw | medRxiv | No explicit explainable AI methodology |
| N/A | Correlation between measured parameters of risk and prognosis in subjects with chronic periodontitis | medRxiv | No explicit explainable AI methodology |
| N/A | Recovering Mandibular Morphology after Disease with Artificial Intelligence | medRxiv | No explicit explainable AI methodology |
| N/A | Association Between Diabetes Mellitus and Endodontic Pathosis | medRxiv | No explicit explainable AI methodology |
| N/A | Geographic patterns of the number of root canals in permanent molars. A Systematic Review | medRxiv | Not an original research article |
| N/A | Effect of laser assisted local anesthesia in single-visit root canal treatment for mandibular molar teeth with acute irreversible pulpitis | medRxiv | No explicit explainable AI methodology |
| N/A | Where do Brazilian dental students seek information about COVID-19? | medRxiv | No explicit explainable AI methodology |
| N/A | Emergency and Sequelae Management of Traumatic Dental Injuries: A Quality Assessment of Clinical Practice Guidelines | medRxiv | Not an original research article |
| N/A | Effects of nasogastric tube on oral microbiome among long-term care patients | medRxiv | No explicit explainable AI methodology |
| N/A | Is Standard Personal Protective Equipment Effective Enough To Prevent COVID-19 Transmission During Aerosol Generating Dental, Oral and Maxillofacial Procedures? A Systematic Review | medRxiv | No explicit explainable AI methodology |
| N/A | Tooth Loss, Patient Characteristics, and Coronary Artery Calcification | medRxiv | No explicit explainable AI methodology |
| N/A | Detecting salivary host-microbiome RNA signature for aiding diagnosis of oral and throat cancer | medRxiv | No explicit explainable AI methodology |
| N/A | Systematic Review and Meta-Analysis on the Effect of Self-Assembling Peptide P11-4 on Initial Caries Lesions | medRxiv | Not an original research article |
| N/A | Systematic Review and Meta-Analysis on the Effect of Self-Assembling Peptide P11-4 on Initial Caries Lesions | medRxiv | Not an original research article |
| N/A | Exploring the use of preprints in dentistry | medRxiv | No explicit explainable AI methodology |
| N/A | Fallow time determination in dentistry using aerosol measurement | medRxiv | No explicit explainable AI methodology |
| N/A | Saliva as a Candidate for COVID-19 Diagnostic Testing: A Meta-Analysis | medRxiv | No explicit explainable AI methodology |
| N/A | Assessing the impact of dental and periodontal statuses on the salivary microbiome: a global oral health scale | medRxiv | No explicit explainable AI methodology |
| 10.4103/JMHIT.JMHIT_37_25 | Speakers’ Presentations | Ovid | No explicit explainable AI methodology |
| 10.1093/ejo/cjaf054. | Diagnostic accuracy of an artificial intelligence-based software in detecting supernumerary and congenitally missing teeth in panoramic radiographs. | Ovid | No explicit explainable AI methodology |
| 10.1097/JS9.0000000000002505. | A review of ChatGPT in medical education: exploring advantages and limitations. | Ovid | Not an original research article |
| 10.4103/jioh.jioh_162_24 | A Narrative Review in Application of Artificial Intelligence in Forensic Science: Enhancing Accuracy in Crime Scene Analysis and Evidence Interpretation | Ovid | Not an original research article |
| 10.1111/tops.12737 | Understanding Human Cognition Through Computational Modeling | Ovid | No explicit explainable AI methodology |
| 10.4103/jomfp.jomfp_448_23 | Narrative review on artificially intelligent olfaction in halitosis | Ovid | Not an original research article |
| 10.1097/HEP.0000000000000580 | The Liver Meeting: Boston, Massachusetts Nov 10-14, 2023 | Ovid | Not related to dentistry/oral health |
| 10.1177/03000605221135147 | Automatic detection of the mental foramen for estimating mandibular cortical width in dental panoramic radiographs : the seventh survey of the Tromsø Study (Tromsø7) in 2015–2016 | Ovid | No explicit explainable AI methodology |

(n = 81)
