# Supplementary material for: Explainable Artificial Intelligence in Dentistry: A Systematic Review of Its Trust and Translation
Source: Int Dent J. 2026 May 25;76(4):109626. doi: 10.1016/j.identj.2026.109626 (PMC13223827; doi:10.1016/j.identj.2026.109626)
Supplement: Supplementary file 4 — Supplementary Table 2: Detailed data extraction of included studies. [file mmc4.docx]

**Supplementary Table 2.** **Detailed data extraction of included studies**.

| **Bibliographic Information** | **Study Characteristics** | **Dental Domain** | **AI Model** | **XAI Method** | **Dataset Details** | **Evaluation Metrics** | **Clinical Relevance** | **Limitations Reported** |
| --- | --- | --- | --- | --- | --- | --- | --- | --- |
| Adnan N et al. (2024) ^17^ | Validation study using intraoral photographs; comparison of AI model vs. junior dentists; hospital-based (Aga Khan Univ., Karachi). | Cariology (dental decay detection). | YOLOv5s CNN (primary model); Detection Transformer (DeTR) for comparison. | EigenCAM saliency maps (visual heatmaps for caries localization). | 7,465 intraoral photographs from pediatric and adult patients; included both primary and permanent dentitions; split into training, validation, and test sets (details: 5,222 training, 1,121 validation, 1,122 test). | YOLOv5s: Precision 90.7%, Sensitivity 85.6%, F1-score 88.0%, ROC AUC 0.79; Junior dentists: Precision 83.3%, Sensitivity 64.1%, F1-score 72.4%, ROC AUC 0.76; AI consistently outperformed human raters in sensitivity. | Developed smartphone-based app provides caries detection comparable or superior to junior dentists; potential for scalable, population-level caries index assessment, especially in low-resource settings without routine dental care. | Limitations: Dataset limited to one institution (generalizability concerns); variations in image quality and lighting may affect performance; only compared AI to junior dentists (not senior specialists); external validation not performed. |
| Dai et al. (2024) ^20^ | Longitudinal, multi-cohort observational analysis developing LP-Micro predictive framework for oral and systemic health outcomes | Pediatric dentistry (ECC prediction); links to systemic conditions (bariatric cohort, BMI outcomes) | Ensemble of ML/DL models (RF, XGBoost, SVM, NN, LSTM, GRU, CNN-GRU) integrated via LP-Micro framework with polynomial group lasso feature selection | Permutation-based feature importance (PermFit) with p-value significance testing for taxa/timepoints | VicGen cohort: 134 children, 6 visits (3–60 months), ECC as outcome; Bariatric cohort: 120 adults, 4–6 visits, BMI as outcome; data = 16S rRNA microbiome sequencing | Improved predictive performance vs baseline models; higher AUC, accuracy, sensitivity, specificity (exact values varied by dataset and model); longitudinal accumulation improved ECC prediction | Identified Streptococcus mutans abundance at 39 months as strongest ECC predictor; demonstrated feasibility of longitudinal microbiome ML for personalized caries prevention | Small pediatric cohort size; class imbalance at timepoints; limited external validation in independent dental cohorts; high computational demand of framework |
| Ikeda T et al. (2022) ^31^ | Prospective longitudinal study; 61,883 community-dwelling older adults (≥65 years), 3-year follow-up; Japan Gerontological Evaluation Study (JAGES) survey data | Geriatric/Oral health (oral health parameters such as number of teeth, chewing/eating difficulty, dry mouth included as predictors) | Random forest with Boruta feature selection; XGBoost (primary predictive model); logistic regression as comparator | SHAP (Shapley Additive Explanations) for feature attribution and interpretability | Dataset: JAGES nationwide survey linked longitudinally (2013–2016); predictors included oral, medical, psychosocial, and functional variables; outcome = incidence of self-reported falls | XGBoost model achieved Accuracy 0.88, F1-score 0.89, AUC 0.88 with resampling; SHAP identified top features including oral health and physical activity | Oral health variables were identified as significant contributors to fall risk; findings support integration of oral health in systemic health risk prediction and geriatric care planning | Limitations: outcome based on self-reported falls (risk of recall/reporting bias); no external validation beyond JAGES dataset; limited generalizability to non-Japanese populations; potential unmeasured confounding despite rich predictors |
| Dai Y et al. (2025) ^21^ | Longitudinal, multi-cohort study. VicGen cohort: 134 children with 6 saliva microbiome samples over 5 years (ECC outcome at 5 years). Bariatric surgery cohort: 120 adults with 6 fecal microbiome samples across 12 months (BMI outcome). Simulated data also used to benchmark. | Pediatric dentistry (focus on early childhood caries via oral microbiome trajectories). | Machine learning: Lasso regression, Random Forest, XGBoost, SVM, Neural Networks (LSTM, GRU, CNN-GRU). Ensemble models tested for prediction. | Polynomial group lasso for feature selection; permutation feature importance with p-values for interpretability. | VicGen: 134 children, 6 timepoints (6–60 months), ECC as binary outcome. Bariatric cohort: 120 adults, BMI trajectory. External validation via simulation. | AUC up to 0.74 for ECC prediction (VicGen, 48 months); Accuracy ~70%; ensemble models improved MSE and PCC compared to baselines; identified predictive taxa trajectories (e.g., Streptococcus mutans). | Identified critical taxa and timepoints (notably S. mutans abundance at 39 months) predictive of ECC, enabling earlier preventive interventions; methodology adaptable to other oral/medical microbiome-based risk predictions. | Sample size relatively small (n=134 children), risk of overfitting; ECC diagnosis only at 5 years, may miss earlier disease onset; model generalizability across populations not yet validated; requires integration with additional biomarkers for clinical deployment. |
| Parola M et al. (2024) ^27^ | Longitudinal, multi-cohort study. VicGen cohort: 134 children with 6 saliva microbiome samples over 5 years (ECC outcome at 5 years). Bariatric surgery cohort: 120 adults with 6 fecal microbiome samples across 12 months (BMI outcome). Simulated data also used to benchmark. | Pediatric dentistry (focus on early childhood caries via oral microbiome trajectories). | Machine learning: Lasso regression, Random Forest, XGBoost, SVM, Neural Networks (LSTM, GRU, CNN-GRU). Ensemble models tested for prediction. | Polynomial group lasso for feature selection; permutation feature importance with p-values for interpretability. | VicGen: 134 children, 6 timepoints (6–60 months), ECC as binary outcome. Bariatric cohort: 120 adults, BMI trajectory. External validation via simulation. | AUC up to 0.74 for ECC prediction (VicGen, 48 months); Accuracy ~70%; ensemble models improved MSE and PCC compared to baselines; identified predictive taxa trajectories (e.g., Streptococcus mutans). | Identified critical taxa and timepoints (notably S. mutans abundance at 39 months) predictive of ECC, enabling earlier preventive interventions; methodology adaptable to other oral/medical microbiome-based risk predictions. | Sample size relatively small (n=134 children), risk of overfitting; ECC diagnosis only at 5 years, may miss earlier disease onset; model generalizability across populations not yet validated; requires integration with additional biomarkers for clinical deployment. |
| Farook TH et al. (2025) ^32^ | In vitro experimental diagnostic accuracy study; mandibular molars with standardized cavity preparations. | Restorative dentistry / caries diagnosis. | 3D Convolutional Neural Network (3D-CNN) with 3 convolutional + 2 fully connected layers. | Saliency maps (heatmaps) used to visualize areas influencing classification. | 56 cavity preparations on mandibular molars (ICDAS codes 3–6), prepared by 2 operators; 3D intraoral scanner used for acquisition. | Accuracy: 94.44% for ICDAS classification; 66.67% for operator differentiation. Dice Similarity Coefficient (DSC): >0.90; Hausdorff distance: ~0.13 ± 0.10 mm. | Provided reliable automated classification of caries depth across ICDAS scores using 3D scans; XAI improved transparency of predictions, supporting unbiased diagnosis and restorative planning. | Small sample size; limited to in vitro setting; only mandibular molars tested; requires validation on larger clinical datasets with broader tooth types and patient variability. |
| Kayadibi I et al. (2025) ^24^ | Retrospective experimental study; CNN-based model (E-mTMCNN) developed and compared with AlexNet, ResNet18, MobileNetV2, SqueezeNet; panoramic radiographs used | Oral and maxillofacial radiology (mandibular third molar detection) | E-mTMCNN (GoogLeNet-based modified CNN); compared against AlexNet, ResNet18, MobileNetV2, SqueezeNet | LIME (Local Interpretable Model-agnostic Explanations) for heatmap-based interpretability | Dataset of 1,317 panoramic radiographs (UESB-based m-TM dataset); split 90% training, 10% testing; expert-labeled ground truth | Accuracy 87.02%, Sensitivity 75%, Specificity 94.73%, Precision 77.68%, F1 75.51%, AUC 0.87 | Provides robust detection of mandibular third molars on panoramic radiographs; supports clinical decision-making and treatment planning in oral/maxillofacial care | Limited dataset size (1,317 images) may affect generalizability; retrospective single-source dataset; further validation on multi-center and larger datasets recommended |
| Dangsungnoen L et al. (2025) ^33^ | User study with 24 participants (12 dental students, 12 data science students); experimental 2×2 factorial design comparing explanation modalities; outcome measures: understandability, trust, willingness to use, task completion time | Oral and maxillofacial radiology; forensic sex classification from panoramic radiographs (orthopantomograms) | DeepToothDuo (EfficientNetB0 backbone); YOLOv5 for tooth localization | OPG-SHAP (tooth-level SHAP visualizations) + Retrieval-Augmented Generation with Gemini 1.5 Flash LLM for textual explanations | Radiographic dataset of orthopantomograms (OPGs); sex labels (male/female); dataset pre-processed via tooth localization and classification pipeline; training/evaluation dataset sizes not explicitly reported in the user-study section (focus on explanation phase) | Dual-modality explanations improved understanding (mean score 7.29 ± 1.12 vs. control 6.12 ± 1.23), trust (3.85 ± 0.53 vs. control 3.41 ± 0.68), and willingness to use (4.16 ± 0.63 vs. control 3.62 ± 0.69); task time increased with textual explanations (416s vs. 348s control) | Demonstrated that combining SHAP visualizations with LLM-generated textual explanations increased user comprehension and trust in AI-driven sex prediction from dental radiographs; highlighted value in forensic dentistry, education, and decision support | Small user sample size (n=24) limits generalizability; no external validation of AI model performance included; explanation evaluation limited to short-term user impressions; task time increased with textual explanations, which may affect practical adoption |
| Angelone F et al. (2025) ^30^ | Prospective study; 79 patients (43 moderate, 36 severe periodontitis); intraoral RGB images; plaque ROIs manually segmented; compared frontal and occlusal views. | Periodontology (periodontitis severity classification). | Random Forest, Naive Bayes, SVM, KNN, Neural Net, CART, LDA. | Partial Dependence Plots (PDPs) for interpretability. | 79 patients; intraoral photographs; features derived from radiomics-inspired texture/color descriptors; split by ROI type (frontal vs. occlusal). | Accuracy: up to 80% (Naive Bayes frontal, blue channel); Sensitivity 57%, Specificity 100%, F1-score 0.73, Balanced Accuracy 0.79. | Demonstrates feasibility of explainable ML on simple intraoral images for non-invasive classification of moderate vs. severe periodontitis; potential for clinical adoption without advanced imaging. | Small dataset (79 patients) may limit generalizability; imbalance across features; manual ROI selection introduces observer variability; external validation not performed. |
| Kamran M et al. (2024) ^18^ | Transformer-based image classification; experimental design with 1,593 dental X-rays; dataset split into training (60%), validation (20%), and testing (20%); data augmentation applied | Caries detection and hypodontia identification | Vision Transformer (ViT, vit-base-patch16-224-in21k pretrained model) | LIME (Local Interpretable Model-Agnostic Explanations), SHAP (SHapley Additive exPlanations), Grad-CAM (Gradient-weighted Class Activation Mapping) | 1,593 periapical and panoramic X-rays (Kaggle + other online repositories); images resized to 224×224; augmentation with flips, rotations, zoom | Accuracy: 96.63%, Precision: 96.60%, Recall: 96.63%, F1-score: 96.57% | Demonstrates high-accuracy, interpretable detection of caries and hypodontia; aids trust and adoption in dental diagnostics | Limited dataset size and sourcing (public repositories only); lack of clinical/real-world validation; no external test set; potential generalizability concerns |
| Milani OH et al. (2025) ^25^ | Special session conference paper; experimental verification study on dental imaging AI. Two case studies: (1) skeletal maturity via CBCT spheno-occipital synchondrosis (SOS) staging; (2) lateral cephalometric radiographs for Class III malocclusion detection. | Orthodontics (skeletal maturity, malocclusion diagnosis) | ResNet18, ResNet34, ResNet50, ConvNeXt, ConvNeXt+Attention (reference model) | Gradient Attention Maps (GAM), Grad-CAM; similarity metrics: Intersection over Union (IoU), Dice Coefficient, Structural Similarity Index (SSIM), Cosine Similarity, Pearson Correlation, Kullback-Leibler (KL) Divergence, Wasserstein Distance; Random Forest classifier to verify model alignment | Dataset 1: CBCT scans for SOS staging. Dataset 2: Cephalometric radiographs for skeletal Class III malocclusion. Both annotated for classification tasks. | Case study 1 (SOS): ConvNeXt+Attention accuracy = 79.9%. Case study 2 (Class III): ConvNeXt accuracy = 90.9%. Verification: AUC = 93.5%, Accuracy = 93.0%, Precision = 91.0%, Recall = 93.0%, F1 = 91.0%. | Provides framework to ensure CNNs are clinically reliable by verifying attention maps align with relevant anatomical regions. Improves transparency, reliability, and safety of AI in orthodontic diagnosis. | Limited to two datasets and imaging tasks; experimental setting only (conference study). Clinical deployment not tested; performance may vary with broader populations and imaging variability. |
| Taskin S et al. (2024) ^28^ | Experimental comparative study using transfer learning. Evaluated multiple CNN architectures fine-tuned for oral disease classification. | General dentistry – classification of common oral diseases (caries, gingivitis, calculus, hypodontia, mouth ulcers, tooth discoloration) from clinical photographs. | MobileNetV2 (best), DenseNet169, ResNet50V2, InceptionV3 (transfer learning, fine-tuning). | Grad-CAM, LIME for saliency-based interpretability. | Dataset: 12,320 images (6 disease categories) sourced from hospitals and Kaggle dental image repository. Undersampled to 7,506 images to balance classes. Split into train (80%), validation (10%), test (10%). | MobileNetV2: Accuracy 89.02%, Precision 89.45%, Recall 89.02%, F1 = 89.13%. DenseNet169: 87.3%. ResNet50V2: 88.9%. InceptionV3: 78.3%. Grad-CAM and LIME maps validated feature attribution for lesions. | Provides interpretable, high-performing AI to support dental diagnostics. Saliency maps improved trust by highlighting regions relevant for disease classification. Potential integration into clinical settings. | Dataset sourced partly from online repositories (Kaggle), may limit generalizability. Risk of bias from data imbalance despite undersampling. No external clinical validation performed. Only six disease classes studied, limiting scope of applicability. |
| Zhu X et al. (2025) ^34^ | Retrospective study; cross-sectional ML model development; NHANES 2009–2014 data; adults ≥30 yrs; n ≈ 10,600 for model training/testing. | Periodontology (periodontitis diagnosis and prognosis, home-based). | Logistic Regression, Random Forest, Histogram Gradient Boosting Trees (HGBT), Support Vector Machine (SVM), 3-layer Multi-Layer Perceptron (MLP). | Feature importance analysis (identifying predictors: age, smoking, diabetes, oral health variables); Recursive Feature Elimination (RFE); dimensionality reduction with PCA and UMAP for misclassified populations. | NHANES 2009–2014, non-radiographic multimodal dataset (demographic, nutrition, medical history, oral health records); training (8,529), validation (1,066), test (1,066). | AUC up to 0.81 (HGBT, SVM, LR, RF); precision ≈0.80; recall ≈0.73; F1-score = 0.74; no overfitting detected; robust performance across models. | Provides non-radiographic, accessible home-based assessment of periodontitis, empowering patients with limited access to dental care; supports early intervention and preventive dentistry. | Dataset limited to U.S. NHANES (generalizability uncertain); cross-sectional design (no temporal prediction); no external validation yet; self-reported oral health data introduces possible bias. |
| Paniagua Rivera K et al. (2024) ^35^ | Preprint (2024). Secondary analysis of experimental dataset aggregated from >200 publications. 233 samples with 17 composite attributes (monomer composition, filler load, depth of cure, conversion) and 7 performance outcomes (Flexural modulus, Flexural strength, Volumetric shrinkage, Shrinkage stress, etc.). | Restorative dentistry – dental composites. | Classification models: KNN, Decision Tree, Random Forest, Logistic Regression, SVM, XGBoost. Regression models: Decision Tree Regressor, Voting Regressor. | Feature importance (permutation-based, variable contribution analysis) to highlight influential predictors (e.g., TEGDMA, BisGMA, UDMA, filler loading, degree of conversion). | Dataset: 233 composites aggregated from >200 published studies; attributes imputed for missing values; multiple cross-validation for model evaluation. | Classification: ROC AUC up to 0.967 (SVM for shrinkage stress), Accuracy up to 0.90. Regression: R² up to 0.93, MAE = 0.49 MPa (Shrinkage stress). Decision Tree outperformed others for flexural strength and shrinkage volume; Voting Regressor best for modulus/shrinkage volume. | Helps optimize material development by predicting mechanical performance, identifying key monomers (TEGDMA, BisGMA, UDMA) and structural features (filler load, depth of cure) most relevant to shrinkage stress and mechanical strength. Supports data-driven composite design. | Limited dataset size (233 samples) relative to ML model requirements; possible overfitting with Decision Trees; lack of external dataset validation; variability in data quality due to reliance on published sources rather than standardized experimental trials. |
| Pham TD (2025) ^22^ | Retrospective diagnostic model development; experimental study design. | Pediatric dentistry, radiology (panoramic images for caries and periapical infection). | Vision–Language Model (hybrid pipeline: CNN for visual features + transformer for text), compared against CNN-only, LSTM, and BERT. | Multimodal explainability: textual description generation (ChatGPT auto-reports) + feature representation pipeline (FRP) + gray-level co-occurrence matrix (GLCM) texture encoding; saliency of combined visual–textual features improves interpretability. | 58 pediatric panoramic radiographs, annotated for caries and periapical infections; compared with human-expert performance; small sample size noted. | Accuracy 90%; Sensitivity 91.7%; Specificity 83.3%; Precision 91.7%; F1-score 0.90; AUC 0.96. | Demonstrates feasibility of combining text and image modalities for AI-assisted pediatric dental diagnosis, improving diagnostic accuracy and interpretability compared with standalone CNN, LSTM, or BERT. | Small dataset (58 cases) limits generalizability; retrospective design; external validation needed; reliance on ChatGPT-generated text could introduce bias. |
| Pham TD (2025) ^23^ | Preprint, experimental study; evaluated text-based classification approach; compared multiple models for pediatric dental disease classification. | Pediatric dentistry, radiology | 1D-CNN, LSTM, BERT; pretrained CNNs (SqueezeNet, GoogLeNet, AlexNet) used for benchmarking. | Proxy XAI via ChatGPT-generated textual descriptions from panoramic radiographs (language-based interpretability). | 70 pediatric panoramic radiographs (training set) and 29 (test set); ground-truth annotation by six dental experts; binary classification: caries vs. periapical infections. | 1D-CNN: ACC 84%, SEN 86.7%, SPE 86.7%, PRE 86.7%, F1=0.84, AUC=0.93; BERT: ACC 76.7%, SEN 83.3%, SPE 66.7%, F1=0.82; LSTM: ACC 56.7%, SEN 75%, SPE 41.7%, F1=0.62; Pretrained CNNs performed worse than text-based models. | Demonstrated that language-based models using generated text descriptions can outperform CNN-only approaches; interpretable and potentially scalable for pediatric diagnostics. | Small dataset size; limited external validation; proxy XAI depends on accuracy of text generation; results may not generalize to larger or more diverse populations. |
| Motmaen I et al. (2024) ^29^ | Retrospective study; 1,184 patients, 26,956 single-tooth panoramic images (annotated extraction vs. preservation). Dataset: training 17,874, validation 4,784, test 4,298. Comparison with 5 dentists. | Oral and maxillofacial surgery; decision support for tooth extraction planning from panoramic radiographs. | ResNet-50 pretrained on ImageNet for binary classification (extraction vs. preservation). | CAMERAS (Class Activation Mapping with Resolution Adaptive Sampling). | PAN radiographs from clinical archive (2011–2021), 1,184 patients, 26,956 teeth. Cropped and annotated per tooth; evaluated at multiple cropping margins (2%, 5%, 10%). | AI model (2% crop): Accuracy 0.834, Sensitivity 0.797, Specificity 0.843, Precision 0.564, F1 = 0.661, ROC-AUC = 0.901, PR-AUC = 0.749. Comparison: dentists ROC-AUC = 0.797, PR-AUC = 0.589. | AI surpassed dentists in tooth extraction prediction; CAMERAS provided interpretable heatmaps highlighting relevant radiographic regions; potential as decision-support system in dentistry to reduce diagnostic errors. | Retrospective single-center dataset; class imbalance (19.1% extraction prevalence); reliance on radiographic features without clinical context; limited external validation; explainability limited to CAMERAS. |
| Devlin H et al. (2021) ^19^ | Randomized controlled study; 23 dentists (11 control, 12 experimental); each assessed 24 bitewing radiographs containing 65 enamel-only proximal caries lesions and 241 healthy proximal surfaces (gold standard: expert panel). | Radiology / Caries detection | Proprietary AI model (AssistDent®); ML-based system for detection prompts. | Visual saliency-like cues: AI generated on-screen prompts (highlighting possible caries sites) → interpretable by clinicians as localized heatmap indicators. | Dataset: 24 standardized bitewing radiographs, annotated by expert panel; 65 enamel-only proximal caries lesions, 241 healthy surfaces. | Sensitivity: 75.8% (AI-assisted) vs 44.3% (control); Specificity: 85.4% (AI-assisted) vs 96.3% (control); Statistically significant differences (p < 0.01). | AI assistance increased sensitivity by 71% for enamel-only proximal caries detection but reduced specificity by 11%. Supports preventive dentistry by improving early detection. | Small sample size (23 dentists, 24 images); single set of radiographs may limit generalizability; modest specificity reduction; performance dependent on quality of gold-standard annotations. |
| Lee J et al. (2024) ^26^ | Retrospective in-silico diagnostic study; cephalometric data from 151 patients (101 initial cohort, 50 validation cohort); diagnostic task = classify facial deformities from cephalometric measurements | Oral & Maxillofacial Surgery (cephalometric diagnosis of jaw deformities) | Large Language Models: LLAMA-2 (7B, 13B, 70B), GPT-3.5, GPT-4, Gemini-Pro; baselines: SVM, Random Forest, XGBoost, MLP | Prompt-based reasoning; chain-of-thought (structured text interpretation of cephalometric values); interpretable textual output | Cephalometric measurements (SNB Angle, Facial Angle, Mandibular Unit Length) converted into textual input; 151 cases total split into training/testing; no external datasets | Balanced accuracy up to 67.7% (LLAMA-2-13B, subsequent cohort); F1-score up to 67.5%; machine learning baselines performed lower; larger LLMs saturated performance with fewer examples | Highlights feasibility of LLMs for automated cephalometric diagnosis; enhances interpretability via textual reasoning; potential tool to support trainees and clinicians in identifying jaw deformities | Small dataset (n=151), limited diversity; results not generalizable; lacks clinical validation; interpretability depends on prompt design; LLMs not yet reliable for autonomous diagnosis |

(n = 19)
