# Supplementary material for: Explainable Artificial Intelligence in Dentistry: A Systematic Review of Its Trust and Translation
Source: Int Dent J. 2026 May 25;76(4):109626. doi: 10.1016/j.identj.2026.109626 (PMC13223827; doi:10.1016/j.identj.2026.109626)
Supplement: Supplementary file 5 — Supplementary Table 3: Detailed explanations for quality assessment of included studies. [file mmc5.docx]

**Supplementary Table 3.** **Detailed explanations for quality assessment of included studies.**

| **Bibliographic Information** | **QUADAS-2 ^a^** | | | | | **PROBAST ^b^** | | | | **Overall**  **Risk of Bias ^c^** |
| --- | --- | --- | --- | --- | --- | --- | --- | --- | --- | --- |
|  | **Patient Selection** | **Index Test** | **Reference Standard** | **Flow & Timing** | **Applicability Concerns** | **Participants** | **Predictors** | **Outcomes** | **Analysis** |  |
| Adnan N et al. (2024) ^17^ | High | Low | High | Low | High | High | Low | High | High | High |
|  | Single-center hospital dataset; possible spectrum bias (images only from one institution). | Index test (YOLOv5s CNN, DeTR) applied consistently and blinded to comparator performance. | Junior dentists used as reference standard, not gold-standard specialists; may bias comparative accuracy. | All patients/ images analyzed; no major exclusions after enrollment. | Limited generalizability beyond one geographic and institutional setting. | Only patients from Aga Khan University, Karachi; not representative of broader populations. | Predictors (image-level caries features) were clearly defined and consistently applied. | Outcome definition (caries vs. healthy) relied on junior dentists, not expert gold standard. | External validation absent; no calibration for different populations; possible overfitting | Especially in participants and outcome domains; limited external validity. |
| Dai et al. (2024) ^20^ | Low | Low | Low | Low | Fair | High | Low | Low | High | Fair |
|  | Multi-cohort data, clear inclusion/exclusion, though limited to children and bariatric adult cohorts | ML/DL models consistently applied; XAI method (PermFit) transparently described | Outcomes (ECC diagnosis, BMI) clinically validated and well defined | Longitudinal follow-up; some attrition acknowledged | Generalizability limited to specific populations | Small pediatric cohort, potential selection bias | Microbiome features and covariates clearly defined | Outcomes clinically relevant and standardized (ECC, BMI) | Possible overfitting due to small sample size; limited external validation | Generally robust methods, but limited generalizability and risk of overfitting |
| Ikeda T et al. (2022) ^31^ | Low | Low | Low | Low | Low | Low | Low | High | Low | Fair |
|  | Large, representative community-dwelling older adult cohort from JAGES survey; clear inclusion criteria | ML models (RF, XGBoost, logistic regression) described, no selective reporting | Outcome (falls) clearly defined, though self-reported | 3-year longitudinal follow-up, consistent flow | Predictors and outcomes relevant to oral/systemic health | Participants well-defined, large sample | Predictors (oral, medical, psychosocial) well-measured | Falls were self-reported, risk of recall bias | Appropriate ML methods, SHAP for interpretability, internal validation performed; no external validation | Mainly due to reliance on self-reported outcome and limited generalizability |
| Dai Y et al. (2025) ^21^ | Low | Low | Low | Low | Low | Low | Low | Low | Fair | Low |
|  | Prospective VicGen and bariatric cohorts, well-described inclusion; but modest sample size. | ML models trained with clear specification; XAI applied systematically. | ECC diagnosis and BMI trajectory are accepted reference outcomes. | Longitudinal sampling and follow-up appropriate, consistent timing. | Limited generalizability (VicGen specific, small n); otherwise relevant. | Pediatric and adult cohorts clearly defined; may not generalize globally. | Microbiome trajectories robustly defined; interpretable feature selection. | ECC diagnosis (binary) and BMI well-defined, clinically relevant. | Multiple models compared; ensemble + resampling used; small sample may risk overfitting. | Overall robust, but modest cohort size and generalizability issues raise caution. |
| Parola M et al. (2024) ^27^ | High | Fair | High | Fair | High | High | Fair | Fair | High | High |
|  | Convenience sampling, small dataset of 567 images, risk of spectrum bias | DL and IDL methods described clearly, but case-based reasoning subjectivity may influence results | No gold-standard histopathology labels for all lesions; many labeled from imperfect/heterogeneous images | No longitudinal follow-up; cross-sectional dataset only | Dataset from limited source, imperfect images; may not generalize to broader populations | Small, single-source dataset limits representativeness | Predictors based on image features + prior knowledge; may not capture all clinical predictors | Outcome defined as lesion classification, but not always biopsy-confirmed | Limited external validation; ensemble metrics reported but with potential overfitting | Risk due to dataset size, patient selection, and lack of robust validation |
| Farook TH et al. (2025) ^32^ | High | Low | Low | Low | High | High | Low | Low | High | High |
|  | Limited to in vitro selection of 56 prepared molars, not representative of clinical population. | 3D-CNN applied consistently with prespecified criteria. | ICDAS classification used as reference standard, widely validated. | All samples processed and evaluated in a uniform timeframe. | Applicability limited, as results from in vitro prepared molars may not generalize to real patients. | Only standardized molar samples, not patient-level data. | Predictors (3D scan features) clearly defined and consistently measured. | Outcomes (ICDAS classification) valid and clinically relevant. | Small sample size, risk of overfitting, no external validation; results may not be robust. | Concerns due to sample size, in vitro design, and lack of generalizability. |
| Kayadibi I et al. (2025) ^24^ | Low | Low | Low | Limited | Low | High | Low | Low | High | Fair |
|  | Retrospective dataset clearly defined, but only single institution | E-mTMCNN and comparators prespecified, XAI method applied | Expert-labeled ground truth used as standard | Retrospective design, limited details on temporal consistency | Relevant dental population and task | Limited to 1,317 images from single institution, generalizability concerns | Predictors (image features) appropriate and pre-specified | Outcomes (presence/absence of mandibular third molar) clearly defined | Relatively small dataset, potential overfitting despite cross-validation; limited external validation | Strong internal validity, but concerns with external generalizability and dataset size |
| Dangsungnoen L et al. (2025) ^33^ | High | Low | High | Low | High | High | Low | High | High | High |
|  | Convenience sample (24 students: 12 dental, 12 data science) not representative of real-world patients | AI model (DeepToothDuo + SHAP + Gemini RAG) clearly described, explanations evaluated consistently | No independent gold standard comparator; task was user evaluation of explanations, not diagnostic accuracy against clinical ground truth | All participants completed tasks in controlled setting with uniform timing | Study evaluated user understanding/trust rather than clinical diagnostic performance; limited generalizability to real dental practice | Very small and non-representative user sample | Predictors (AI visual/text explanations) clearly defined | Outcome not diagnostic accuracy but subjective measures (understanding, trust, willingness to use) | No external validation; descriptive statistics only; no robustness checks | Overall high risk of bias due to non-clinical design, small sample, and subjective outcomes |
| Angelone F et al. (2025) ^30^ | High | Low | High | Low | Fair | High | Low | High | High | High |
|  | Small convenience sample (79 patients) with unclear representativeness; possible selection bias. | Index test (ML models) clearly described, but hyperparameter tuning and thresholds may introduce bias. | No established gold standard; severity classification based only on intraoral ROI features, not validated against full periodontal exam. | All patients processed consistently; no differential verification. | Use of intraoral RGB images may not fully reflect standard diagnostic workflow in periodontology. | Small and single-center cohort; external validity questionable. | Predictor features well defined (radiomic descriptors), but manual ROI introduces variability. | Severity label (moderate vs. severe) not linked to standardized clinical measures. | Small dataset, lack of external validation, possible overfitting despite multiple algorithms. | Driven by limited sample, non-standard outcome definition, and lack of external validation. |
| Kamran M et al. (2024) ^18^ | High | Low | High | High | High | High | Low | High | High | High |
|  | Dataset from public repositories, not representative of clinical patient populations | Index test (ViT model) clearly described and applied consistently | No clinical ground truth confirmation; labels from public datasets only | Unclear temporal sequence; no external validation; retrospective | Dataset not representative; limited applicability to clinical practice | Convenience sample from online repositories; not clinical cohorts | Predictors (image features) clearly defined and preprocessed | Outcomes (caries, hypodontia) not validated against clinical gold standard | Limited dataset, no external validation, possible overfitting | Multiple biases due to dataset limitations, lack of external validation, and representativeness concerns |
| Milani OH et al. (2025) ^25^ | High | Low | Fair | Limited | Fair | High | Low | Fair | Fair | Fair |
|  | Datasets were limited (CBCT SOS staging and cephalometric radiographs) and selection details were not fully described. | AI models and attention verification methods (GAM, Grad-CAM) clearly defined. | Reference standards (SOS staging, Class III diagnosis) based on dataset labels; not explicitly validated by multiple clinicians. | Flow and timing of imaging/annotation processes not fully described. | Orthodontic imaging domain is clear, but generalizability to broader dental populations uncertain. | Participants not fully representative (single-task datasets, limited scope). | Predictors (CNN-extracted features, attention maps) clearly defined. | Outcomes (classification accuracy, alignment verification) clinically relevant but surrogate for patient-level outcomes. | Analysis included strong verification metrics (AUC, F1, similarity indices) but external validation lacking. | High risk of bias due to limited datasets, unclear patient representativeness, and absence of external clinical validation. |
| Taskin S et al. (2024) ^28^ | High | Low | High | Limited | High | High | Low | Fair | High | High |
|  | Dataset combined from Kaggle and hospital images, potential selection bias and lack of prospective sampling. | Index test (MobileNetV2 and other CNNs) was clearly described, but risk exists due to overfitting from transfer learning and undersampling. | No established clinical reference standard; labels derived from datasets may vary in reliability. | Unclear consistency of timing and labeling across sources; retrospective data limits assessment. | Applicability limited since dataset may not represent real-world patient variability. | Participants not representative (images from mixed online and hospital sources). | Predictors (image features) appropriate and pre-specified via CNN fine-tuning. | Outcome defined as disease classification into six categories; limited external validation. | Risk of overfitting due to undersampling and no independent clinical dataset for testing. | Significant concerns regarding dataset representativeness, reference standard validity, and external applicability. |
| Zhu X et al. (2025) ^34^ | Low | Low | High | High | Low | Low | Low | High | Low | Fair |
|  | Population derived from large representative NHANES dataset; clear inclusion criteria. | ML algorithms prespecified, applied consistently. | Periodontitis status derived from NHANES periodontal charting and self-reports; no gold standard clinical re-exam. | Cross-sectional design; no follow-up; no longitudinal validation. | U.S. NHANES generalizable to U.S. adults, but limited transferability to other populations. | Adults ≥30 years included, sample representative of national survey. | Predictors well-defined, mostly objective (demographic, medical, oral health variables). | Outcomes based on NHANES definitions, not externally validated against standardized clinical diagnosis. | Used proper resampling (train/validation/test split), but no external dataset; risk of overfitting reduced but not eliminated. | Strength in sample size and methods, but limitations from outcome reference standard and lack of external validation. |
| Paniagua Rivera K et al. (2024) ^35^ | High | Low | High | High | High | High | Low | Low | High | High |
|  | No patient selection; dataset compiled from literature, not clinical sampling. | AI models (KNN, SVM, Decision Tree, RF, Voting Regressor) were clearly described and consistently applied. | No gold-standard experimental validation; outcomes aggregated from heterogeneous studies. | Temporal and procedural consistency not applicable due to secondary aggregated dataset. | Generalizability to real-world composites limited; models based on published study data only. | Participants not clinical patients, but material samples from >200 publications. | Predictors (monomers, filler load, degree of conversion, etc.) were well-defined and reproducible. | Outcomes (flexural modulus, strength, shrinkage stress, etc.) clearly specified. | Risk of overfitting; limited external validation; possible publication bias in aggregated dataset. | Secondary data source, lack of clinical population, and no external validation. |
| Pham TD (2025) ^22^ | High | Low | High | Limited | Fair | High | Low | Fair | High | High |
|  | Retrospective convenience sample of 58 pediatric radiographs; small size, possible selection bias. | Index test (vision–language AI model) described clearly and applied consistently. | Ground truth annotations relied on limited expert labeling; no standardized multi-expert consensus. | Retrospective design; unclear timing of imaging vs annotation; no prospective flow. | Model trained on a narrow dataset, limits generalizability across pediatric populations. | Small, non-representative sample of pediatric patients. | Ppredictors (image + text features) well described, though limited in scope. | Outcomes (caries vs periapical infection) are clinically relevant but defined on limited gold-standard validation. | Analysis limited by very small dataset; no robust external validation; risk of overfitting. | Overall risk due to small dataset, selection bias, and weak reference standard. |
| Pham TD (2025) ^23^ | High | Low | Low | High | High | High | Low | Low | High | High |
|  | Small convenience dataset (70 training, 29 test) may not represent broader pediatric population. | Index tests (1D-CNN, LSTM, BERT, pretrained CNNs) clearly described and applied consistently. | Ground-truth annotations by six dental experts used as reference standard. | Limited dataset split; potential bias due to small test set and no external validation. | Generalizability to diverse populations and real-world settings uncertain. | Pediatric sample limited in size and diversity. | Predictors (textual descriptions from radiographs) appropriately defined. | Binary outcome (caries vs periapical infection) clinically relevant and well defined. | No external validation; small sample; potential overfitting. | Small dataset, lack of external validation, and generalizability concerns. |
| Motmaen I et al. (2024) ^29^ | High | Low | Fair | Low | Fair | High | Low | Fair | High | High |
|  | Retrospective single-center dataset, potential selection bias in included radiographs. | AI (ResNet-50) and CAMERAS explainability clearly described. | “Ground Truth” based on dentist consensus; may introduce subjectivity. | Consistent annotation and splitting into training/validation/test sets. | Clinical applicability limited, as only radiographic data used without patient context. | Single-center retrospective sample, limited generalizability. | Predictors (radiograph-based features) clearly defined and reproducible. | Binary outcome (extraction vs preservation) reliable but lacks clinical context validation. | Class imbalance, no external validation, single test dataset, potential overfitting. | Retrospective design, dataset limitations, and lack of external validation. |
| Devlin H et al. (2021) ^19^ | Low | Low | Low | Low | Low | Low | Low | Low | Low | Low |
|  | Dentists randomly assigned; representative of target users. | AI prompts applied consistently; no access to gold-standard during assessment. | Expert panel consensus used as reference standard; robust. | All dentists assessed same 24 radiographs under standardized conditions. | Setting (bitewing radiographs, general dentists) aligns with clinical practice. | Participants (23 UK dentists) appropriate for the research question. | Predictors (AI assistance vs no AI) clearly defined and reproducible. | Outcomes (sensitivity, specificity vs gold standard) clearly defined and objectively measured. | Statistical analysis appropriate (comparative metrics, p-values); sample size small but adequate for pilot. | Study well designed but limited by modest sample size and generalizability. |
| Lee J et al. (2024) ^26^ | High | High | High | High | Limited | High | High | High | High | High |
|  | Retrospective dataset, limited size (151 patients), potential selection bias in cohort construction | LLMs not standardized as diagnostic index tests; prompt engineering may influence results | No gold standard diagnostic validation; labels derived from cephalometric thresholds only | Timing between measurements and diagnostic classification not clearly reported | Narrow dataset, limited generalizability to broader populations | Small, single-center dataset, not representative | Predictors limited to 3 cephalometric measurements, may not capture full variability | Outcome = LLM-based classification vs. cephalometric categories; lacks clinical validation | Small sample size, no external validation, possible overfitting; analysis relies on balanced accuracy/F1 only | Insufficient data, limited predictors, lack of external/clinical validation |

(n = 19)
